# Prednisone-hydrogen sulfide releasing hybrid shows improved therapeutic profile in asthma

**DOI:** 10.3389/fphar.2023.1266934

**Published:** 2023-10-12

**Authors:** Ida Cerqua, Elisabetta Granato, Angela Corvino, Beatrice Severino, Danilo D’Avino, Martina Simonelli, Elisa Perissutti, Antonia Scognamiglio, Davida Mirra, Bruno D’Agostino, Giuseppe Caliendo, Antonietta Rossi, Giuseppe Cirino, Chiara Maria Motta, Fiorentina Roviezzo

**Affiliations:** ^1^ Department of Pharmacy, School of Medicine and Surgery, University of Naples Federico II, Naples, Campania, Italy; ^2^ Department of Environmental, Biological and Pharmaceutical Sciences and Technologies, University of Campania Luigi Vanvitelli, Caserta, Campania, Italy; ^3^ Department of Biology, Polytechnic and Basic Sciences School, University of Naples Federico II, Naples, Campania, Italy

**Keywords:** hydrogen sulfide-donor, steroid, airway remodeling, bronchial reactivity, lung inflammation

## Abstract

**Introduction:** Hydrogen sulfide (H_2_S) is emerging as an important potential therapeutic option for respiratory inflammatory diseases. In this study, we investigated the effectiveness of a novel corticosteroid derivative, that is chemically linked to an H_2_S donor, in managing asthma features.

**Methods:** The effects of prednisone (PS), H_2_S donor (4-hydroxybenzamide; TBZ), and their combination (PS-TBZ) have been evaluated *in vitro* and *in vivo*. The *in vitro* experiments were conducted using lipopolysaccharidestimulated J774 macrophages, while the *in vivo* experiments utilizing an experimental asthma model.

**Results:** In the *in vitro* study we found that PS-TBZ exhibited an increased effect compared to the individual parent compounds in modulating the production of inflammatory mediators. TBZ also significantly reduced bronchial contractility and enhanced bronchial relaxation. In the *in vivo* experiments, where we administered PS, TBZ, or PS-TBZ to ovalbumin-sensitized BALB/c mice, we confirmed that PS-TBZ had a significantly better action in controlling airway hyperreactivity as compared to TBZ or PS alone. Moreover, PS-TBZ was more effective in restoring salbutamol-induced relaxation. The immunohistochemistry analysis demonstrated a significant reduction in the production of α-SMA and procollagen III, indicating the efficacy of PS-TBZ in controlling airway remodeling. Moreover, PS-TBZ also promoted epithelial repair, recovery of the bronchial and parenchyma structure and inhibited mucin production.

**Discussion:** In conclusion, PS-TBZ offers an important opportunity to optimize the beneficial impact of corticosteroids on asthma features.

## 1 Introduction

Hydrogen sulfide (H_2_S) is an endogenous gasotransmitter and exerts physiological and pathophysiological effects in several systems (Cirino et al., 2023). Recent research has contributed to elucidating the basic biology of H_2_S in the respiratory system and its relationship with lung disease pathophysiology (Roviezzo et al., 2015; Cirino et al., 2023). The expression of the enzymes Cystathionine-ɣ-lyase (CSE), cystathionine-β-synthase (CBS), and 3-mercapto-sulphurtransferase (3-MST) that catalyze the synthesis of H_2_S (Dilek et al., 2020), has been demonstrated in the airways of different species (Cirino et al., 2023).

Alterations in H_2_S levels has linked with lung disease pathophysiology (Cirino et al., 2023). Indeed, serum H_2_S levels decrease in asthmatic subjects and H_2_S levels reduction is accompanied by lower expression of CSE in ovalbumin (OVA)-sensitized mouse and rat lung tissues (Chen et al., 2009a). On the other hand, CSE deficiency or inhibition of H_2_S biosynthesis by inhibiting CSE activity with propargylglycine has been shown to increase asthma features such as airway reactivity (Chen et al., 2009b). Sodium hydrogen sulfide (NaHS), a fast-releasing H_2_S donor, induces a relaxation response in mouse tracheal and bronchial smooth muscles and diminishes airway hyperreactivity in experimental airway inflammation models (Roviezzo et al., 2015). These pieces of evidence indicate H_2_S as a potential therapeutic option for asthmatic subjects.

Airway remodeling is a challenging issue in lung diseases like asthma, causing irreversible loss of lung function (Hirota and Martin, 2013; Varricchi et al., 2022). It involves structural changes in the airway walls (Hirota and Martin, 2013), which occur due to repeated injury and repair processes (Tan et al., 2022). This phenomenon encompasses alterations in tissue, cellular, and molecular composition, affecting various components, including airway smooth muscle (James et al., 2012), epithelium (Broide et al., 2005), blood vessels (Chetta et al., 2003), and extracellular matrix (Ito et al., 2019). While current therapies can alleviate inflammation, there is no proven treatment to prevent or reverse airway remodeling (Royce and Tang, 2009; Hirota and Martin, 2013). Airway remodeling is often associated with long-term airway inflammation (Hirota and Martin, 2013), but it may also be present to a similar extent in the airways of children with asthma, emphasizing the need for early and targeted therapeutic interventions (Baena-Cagnani et al., 2007). Corticosteroids, a key component of anti-inflammatory asthma therapy (Barnes, 2006), effectively manage airway narrowing and asthma attacks (Alangari, 2014). However, their impact on the aspects of remodeling leading to a decline in lung function remains limited (Varricchi et al., 2022). There is compelling evidence suggesting that H_2_S may have the potential to alleviate airway remodeling (Chen et al., 2009a; Chen et al., 2009b; Guan et al., 2020; Wang et al., 2020).

From a therapeutic perspective, the development of safe H_2_S donors with controllable release and efficient delivery methods to patients is progressing (Magli et al., 2021). H_2_S represents a reductant species because of the oxidation state of the sulfur atom in which is −2; it can also be considered a potent nucleophile at physiological pH values. These features make H_2_S a very reactive acid that can react with many biological molecules. Some evidence reported in the literature suggests that H_2_S also mediates an important oxidative post-translational modification and in particular protein S-sulfuration, generating, for example, S-SH groups on cysteine residues. The low pharmacokinetic profile of sulfide salts limits their use as potential therapeutics and has led to the research into innovative organic H_2_S donors. Many attempts have been made to find effective donor molecules that mimics the slow, gradual, and controlled release of the physiological H_2_S production *in vivo* (Hughes et al., 2009). Some compounds with controllable release rates triggered by different mechanisms (Corvino et al., 2021b) have been identified such as the Lawesson’s reagent and its water-soluble derivative GYY4137 (Rose et al., 2015), N-(benzoylthio) benzamides (Zhao et al., 2011), arylthioamides, such as TBZ, (Martelli et al., 2013)], 1,2,4- thiadiazolidin-3,5-diones (Severino et al., 2018), dithioates, such as 4-hydroxybenzodithioate (HBTA), (Ercolano et al., 2019), and isothiocyanates (Citi et al., 2020). In this study, we have investigated the effectiveness of a novel corticosteroid derivative containing an H_2_S moiety such as TBZ associated to prednisone in controlling asthma-associated airway features.

## 2 Materials and methods

### 2.1 J774 cell culture

The murine monocyte/macrophage J774 cell line was obtained from the American Type Culture Collection (ATTC TIB 67). The cell line was grown in adhesion in Dulbecco’s modified Eagles medium (DMEM) supplemented with glutamine (2 mM, Aurogene Rome, Italy), Hepes (25 mM, Aurogene Rome, Italy), penicillin (100 μ/mL, Aurogene Rome, Italy), streptomycin (100 μg/mL, Aurogene Rome, Italy), fetal bovine serum (FBS, 10%, Aurogene Rome, Italy) and sodium pyruvate (1.2%, Aurogene Rome, Italy) (DMEM completed). The cells were plated at a density of 1 × 10^6^ cells in 75 cm^2^ culture flasks and maintained at 37°C under 5% CO_2_ in a humidified incubator until 90% confluence. The culture medium was changed every 2 days. Before a confluent monolayer appeared, the sub-culturing cell process was carried out. Cells were pre-treated for 2 h in the absence or presence of test compounds (30, 100, and 300 μM) and then stimulated for 24 h with lipopolysaccharide (LPS) (10 μg/mL) from *Escherichia coli*. After 24 h of incubation with LPS, the supernatants were collected for the measurement of prostaglandin (PG)E_2_ (Cayman Chemical, Vinci-Biochem, Vinci, Italy), interleukin (IL)-1β and tumor necrosis factor (TNF)-α (R&D Systems, Aurogene, Rome, Italy) levels by ELISA assay. The nitrite concentration in the samples was measured by the Griess reaction, by adding 100 μL of Griess reagent (0.1% naphthylethylenediamide dihydrochloride in H_2_O and 1% sulphanilamide in 5% concentrated H_2_PO_4_; vol. 1:1; Sigma Aldrich, Milan, Italy) to 100 μL samples. The optical density at 540 nm (OD540) was measured immediately after Griess reagent addition, using ELISA microplate reader (Thermo Scientific, Multiskan GO). Nitrite concentration was calculated by comparison with OD540 of standard solutions of sodium nitrite prepared in a culture medium. IC_50_ analysis for PS, TBZ, and PS-TBZ has been performed with GraphPad 9 software.

#### 2.1.1 Cell viability

Mitochondrial activity, an indicator of cell viability, was assessed by the mitochondrial-dependent reduction of 3-(4,5-dimethylthiazol-2-yl)-2,5-diphenyltetrazolium bromide (MTT; Sigma Aldrich, Milan, Italy) to formazan. Cells were plated to a seeding density of 1.0 × 10^5^ in 96 multiwells. After stimulation with test compounds for 24 h, cells were incubated in 96-well plates with MTT (0.2 mg/mL), for 1 h. The culture medium was removed by aspiration and the cells were lysed in DMSO (0.1 mL). The extent of reduction of MTT to formazan within cells was quantified by the measurement of OD550.

### 2.2 Animals

Female BALB/c (8–9 weeks old, 18–22 g, Charles River, Calco, Italy) were housed in a controlled environment (temperature 21°C ± 2°C and humidity 60% ± 10%) with a 12-h light/dark cycle at the Department of Pharmacy (University of Naples, Italy). All mice were supplied with a standard rodent chow diet and water and acclimatized for 4 days before experiments. All experiments were conducted during daylight according to Italian regulations on the protection of animals used for experiments and other scientific purposes (D.Lgs. 26/2014) and with the European Economic Community regulations (EU Directive 2010/63/EU). Animal studies are reported in compliance with the ARRIVE guidelines (Kilkenny et al., 2010).

### 2.3 *In-vivo* treatments

BALB/c mice were sensitized with a subcutaneous (s.c.) administration of Ovalbumin (OVA, 100 µg) complexed with alum (13 mg/mL) on days 0 and 7 (Cerqua et al., 2022a). On the sixth and seventh days animal groups were pretreated intraperitoneally (i.p.) 30 min before OVA with prednisone (PS, 1 mg/kg, 60 nmol), 4-hydroxy-thiobenzamide (TBZ, 130 nmol), and prednisone-succinate-TBZ (PS-TBZ, 30 nmol). This treatment has been protracted every day up to the 14th day of sensitization.

### 2.4 Bronchial reactivity measurements

Mice were anesthetized with Alfaxan/Xylazine 40 mg/kg injected i.p. and then killed by a vertical middle incision of the thorax to harvest the respiratory system. Right and left bronchi were dissected from the lungs, cut into small rings (1–2 mm), and placed in the isolated organ bath (3 mL) filled with Krebs solution at 37°C (mol/l: NaCl 0.118, KCl 0.0047, MgCl_2_ 0.0012, KH_2_PO_4_ 0.0012, CaCl_2_ 0.0025, NaHCO_3_ 0.025, and glucose 0.01) and oxygenated with a mix of 95% O_2_ and 5% CO_2_. Bronchi were mounted to isometric force transducers (type 7006; Ugo Basile, Comerio, Italy), and connected to a Powerlab 800 (ADInstruments, Italy). Rings were initially stretched until a resting tension of 0.5 g and allowed to equilibrate for at least 30 min, during which the Krebs solution was periodically changed. In each experiment, bronchial rings were first challenged with carbachol (10^−6^ M), until the contraction curve was reproducible, and then the bronchial relaxation was evaluated by performing a cumulative concentration-response curve of salbutamol (10^−5^ M^−3^ × 10^−8^ M) following the carbachol precontraction (Chen et al., 2009a; Chen et al., 2009b).

### 2.6 Measurement of plasma IgE levels

Blood was collected by intracardiac puncture from male and female BALB/c mice using citrate (3.8%) as an anticoagulant. Then plasma was obtained by centrifugation at 12.000 rpm at 4°C for 10 min and immediately frozen at −80°C. Total IgE levels were measured with enzyme-linked immunosorbent assay (ELISA) using matched antibody pairs (BD Biosciences Pharmingen San Jose, CA).

### 2.7 Lung histology and immunohistochemistry

The right lung lobes were harvested from mice and rapidly fixed in 4% formalin. The tissues were embedded in paraffin and sections of 7 μm were cut. The slices were processed to remove paraffin, and following rehydration, hematoxylin-eosin (H&E), Picrosirius red (PSR), Periodic Acid-Schiff plus Alcian Blue (PAS+/AB), and WGA staining were performed. Other slices were used for immunohistochemistry analysis and after rehydration were incubated with 3% hydrogen peroxide (Sigma-Aldrich H1009) for 15 min to quench the endogenous peroxidase activity. Tissue sections were then incubated with 3% BSA (Sigma-Aldrich A9418) for 1 h to reduce non-specific antibody reactions. Sections were incubated overnight at 4°C with primary antibodies diluted in 3% BSA (Anti-Actin, α-Smooth Muscle antibody, 1:200, Sigma-Aldrich A5228; PIIINP, 1:100, Biomatik). After rinsing with phosphate-buffered saline 0.01 M, slides were incubated with Peroxidase Affinity Pure Goat anti-Mouse (Jackson ImmunoResearch, 1:500) for 1 h at room temperature. Colour development was performed using 3,3′-Diaminobenzidine Chromogen Solution (SIGMAFASTTM-DAB). Sections were analyzed using a Leica Microsystem with a magnification of ×20 (H&E, α-SMA, PIIINP, WGA) or 40x (H&E, PRS, PAS+/AB, WGA). To analyze the collagen fibers organization the lung slices were also observed with confocal microscopy Zeiss LSM700 with a magnification of ×40 (Cerqua et al., 2022a).

### 2.8 Statistical analysis

The results are expressed as mean ± S.E.M of n observations, where n represents the number of animals. Statistical evaluation was performed by one-way or two-way ANOVA using GraphPad InStat (Graphpad Software Inc., San Diego, CA) followed by a Bonferroni *post hoc* test for multiple comparisons. Post hoc tests were run only if F achieved *p* < 0.05 and if there was no significant variance in the homogeneity. A *p*-value <0.05 was used to define statistically significant differences between mean values. Grubbs’ test identified outliers.

## 3 Results

### 3.1 TBZ ameliorates the anti-inflammatory effects of PS and modulates the bronchial tone

**FIGURE 1:**
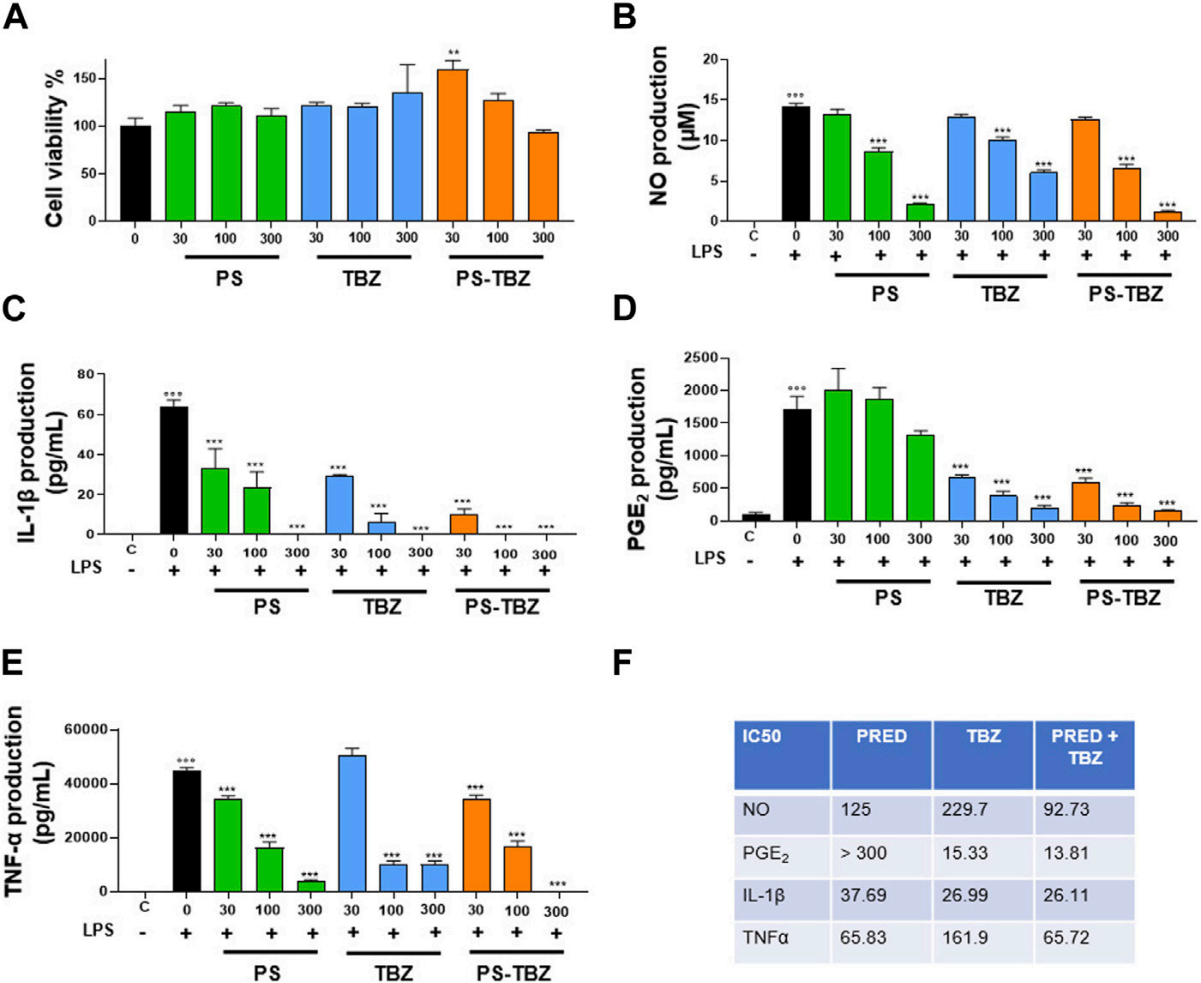
Effect of PS, TBZ, or PS-TBZ on LPS-stimulated murine macrophages: **(A)** Cells were pre-treated for 24 h with PS, TBZ, or PS-TBZ (30–300 µM), and cell viability was evaluated by the mitochondrial-dependent reduction of MTT to formazan. **(B–E):** Cells were pretreated for 2 h with PS, TBZ, or PS-TBZ (30–300 µM) and then stimulated for 24h, with LPS (10 μg/mL). The supernatants were collected for the measurement of nitrite **(B)**, IL-1β **(C)**, PGE_2,_ **(D)**, and TNFα **(E)** levels by ELISA assay. **(F)** Analysis of IC_50_ has been performed with GraphPad 9 software. Values represent means ± S.E.M.; *n* = 3 experiments. Data were analyzed by one-way ANOVA plus Bonferroni *post hoc* test. Statistical significance is reported as follows °°° *p* < 0.001 vs. unstimulated cells, *p < 0.05, **p < 0.01, and ***p < 0.001 vs. LPS alone.

**FIGURE 2:**
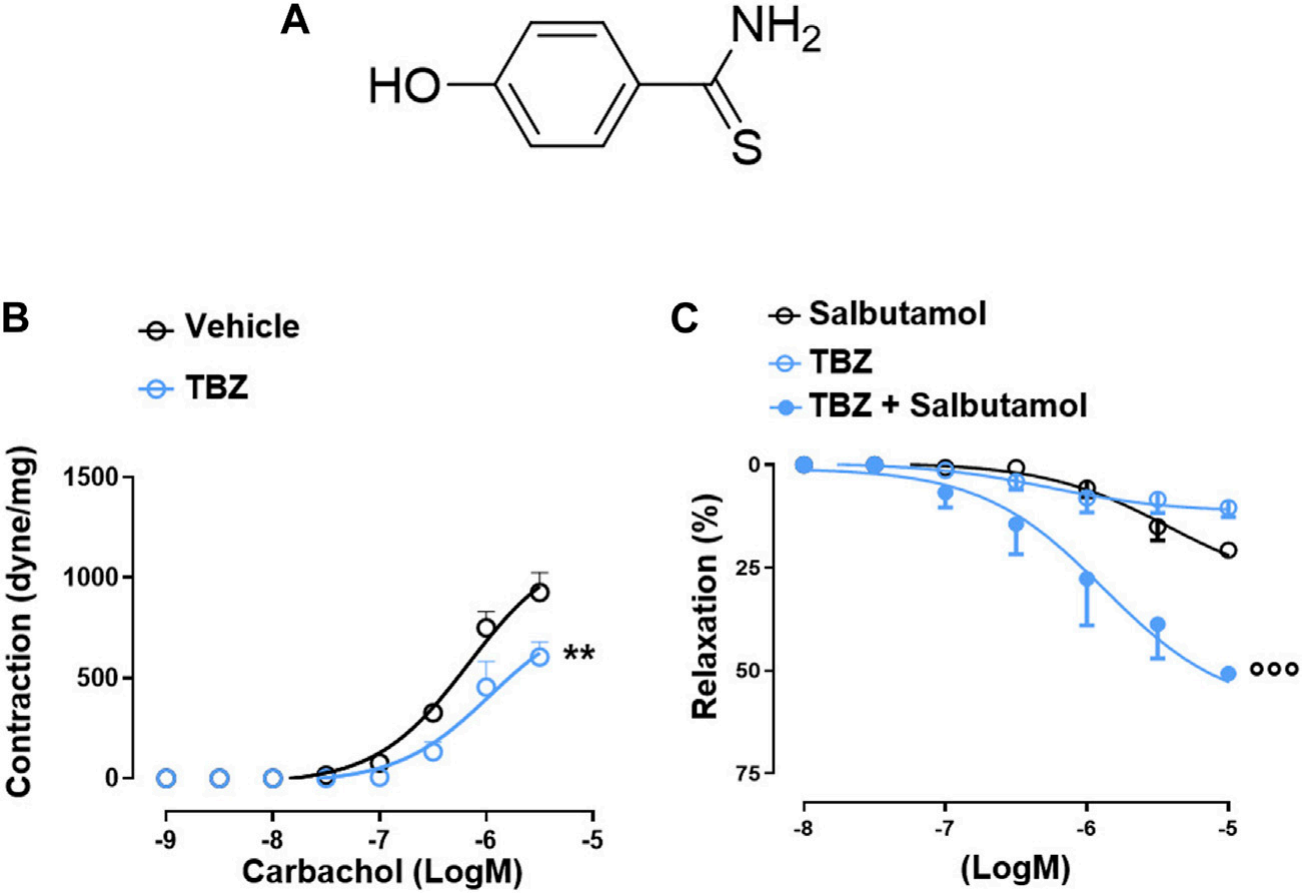
TBZ modulates bronchial tone *in vitro*. Chemical structure of TBZ **(A)**. Bronchi harvested from BALB/c mice were incubated on basal tone with TBZ (30 μM, 45 min) and then stimulated with carbachol **(B)**. Bronchi were stimulated with the β_2_-agonist salbutamol in the presence or absence of TBZ, or with TBZ **(C)**. Values represent means ± S.E.M.; *n* = 6 mice for each group. Data were analyzed by two-way ANOVA plus Bonferroni *post hoc* test. Statistical significance is reported as **p < 0.01 vs*.* vehicle; °°° *p* < 0.001 vs*.* salbutamol.

The pharmacological efficacy of PS-TBZ in controlling inflammation was first evaluated *in vitro* on the macrophage cell line J774 (Figure 1). Both PS and TBZ, at nontoxic concentrations (Figure 1A), exhibited in a concentration dependent manner a concentration-dependent anti-inflammatory activity as indicated by the decreased production of LPS-induced nitric oxide (NO), IL-1β, PGE_2_, and TNF-α (Figures 1B–E). PS and TBZ showed different potency in relation to the different mediators, while PS-TBZ clearly showed a synergic effect (Figure 1F). To note, the combination achieved an effect at concentrations that were significantly lower than those required when parent compounds were administered alone. This means that a certain effect is achieved with smaller moles of the hybrid compound than the parent drug, reducing the dose and, consequently, the side effects. Exposure of bronchial rings to TBZ (Figure 2A) demonstrated its ability to affect also bronchial tone. TBZ inhibited bronchial contraction induced by carbachol (Figure 2B) and potentiated β_2_-receptor mediated bronchorelaxation assessed by exposure of isolate bronchi to salbutamol (Figure 2C).

### 3.2 PS-TBZ reduces airway hyperresponsiveness (AHR) and restores β_2_-mediated relaxation in OVA-sensitized mice

**FIGURE 3:**
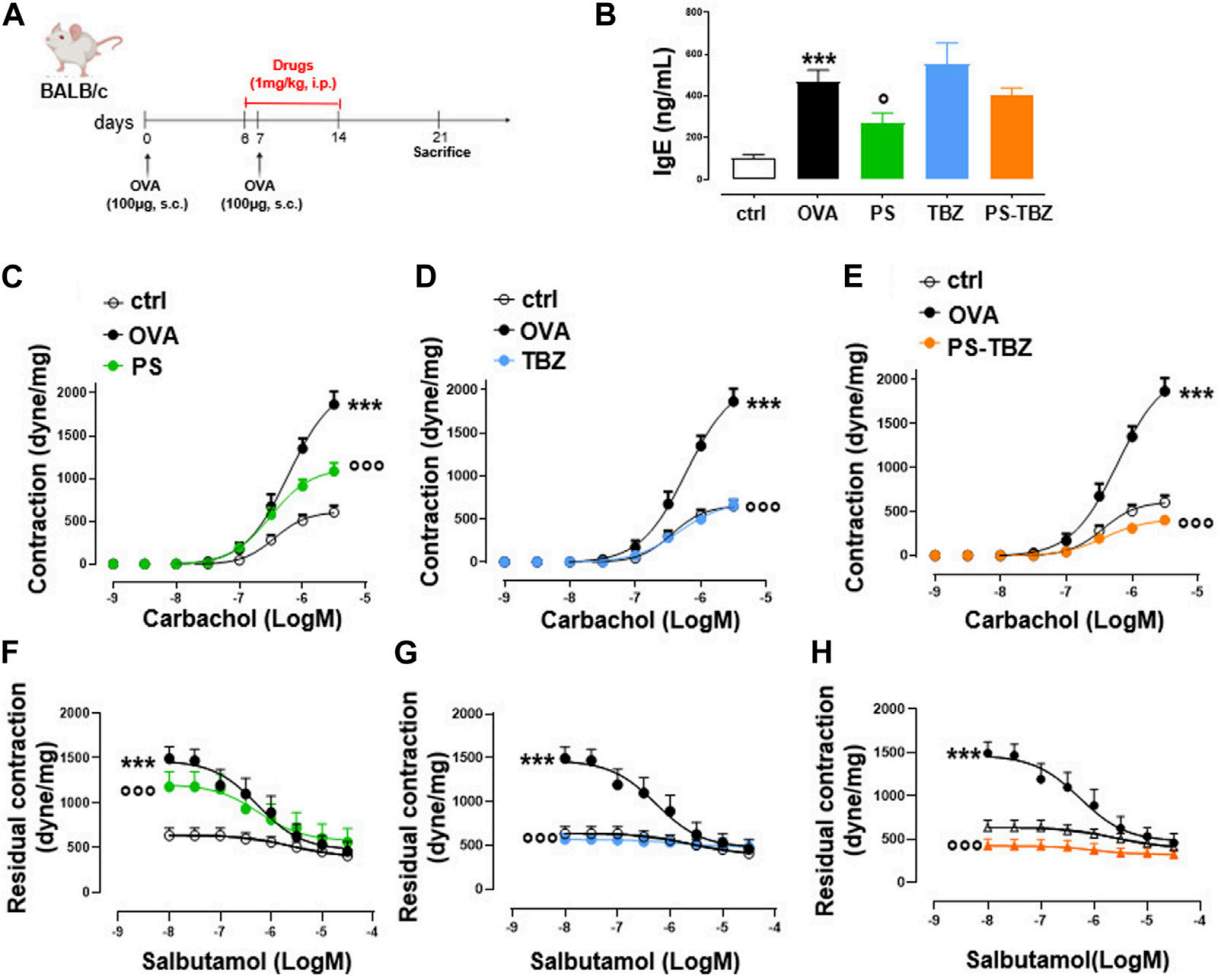
Effect of PS, TBZ, or PS-TBZ in an experimental model of asthma. **(A)**. Mice were sacrificed to measure the plasmatic level of IgE **(B)** and evaluate bronchial reactivity to carbachol **(C–E)** or salbutamol **(F–H)**. Values represent means ± S.E.M.; *n* = 6 mice for each group. Data were analyzed by two-way ANOVA plus Bonferroni *post hoc* test. Statistical significance is reported as ***p < 0.001 vs*.* ctrl; °°° *p* < 0.001, °*p* < 0.05 vs*.* OVA.

The effect of PS-TBZ was evaluated in an experimental model of asthma induced by OVA and compared to the parent compounds PS and TBZ (Figure 3A). Following OVA exposure, an increase in the plasmatic level of IgE occurred (Figure 3B). PS reduced IgE levels, while TBZ and PS-TBZ had no effect. Bronchi harvested from OVA-sensitized mice showed increased reactivity to carbachol (AHR). PS partially decreased AHR (Figure 3C) while TBZ (Figure 3D) and PS-TBZ (Figure 3E) reversed AHR. Although PS-TBZ displayed a similar effect to TBZ on AHR, the association of reducing the dosage of the parent compounds confirms the synergic effect of TBZ with PS (Figure 3E).

Further, the allergic inflammation induced by OVA caused an impairment in β_2_-adrenoceptor mediated relaxation as demonstrated by the significant reduction in relaxant effect elicited by salbutamol expressed (Figures 3F–H). However, this effect is restored by both TBZ (Figure 3G) and PS-TBZ (Figure 3H) treatment, but not by PS alone (Figure 3F).

### 3.3 PS-TBZ induces the recovery of the OVA-induced epithelial damage

**FIGURE 4:**
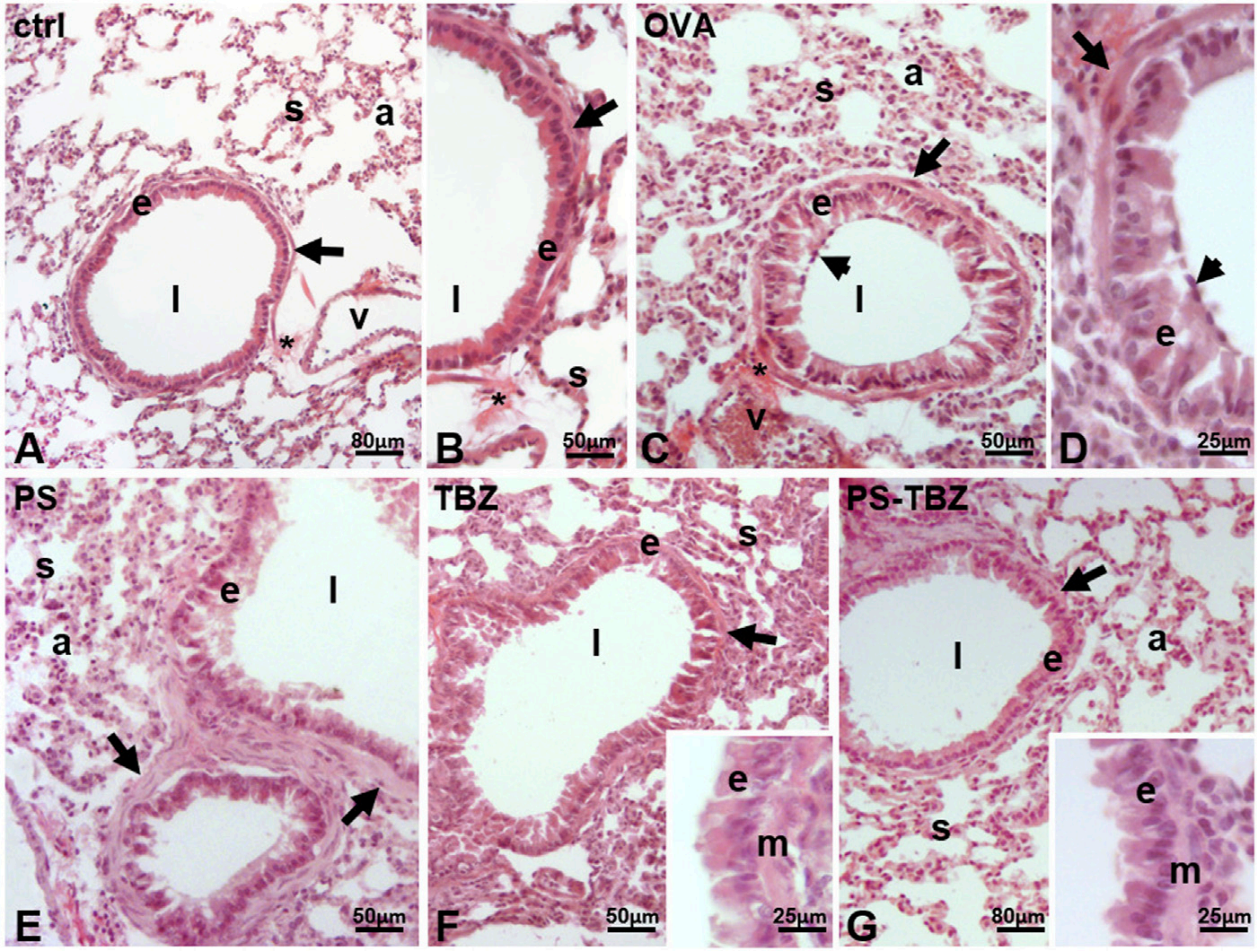
Effects of PS, TBZ, or PS-TBZ treatments on lung structure of sensitized mice. Lungs harvested from control **(A, B)**, OVA- **(C, D)**, PS- **(E)**, TBZ- **(F)**, and PS-TBZ- **(G)** treated mice were stained for Hematoxylin and Eosin **(H, E)** to evaluate the pulmonary structure. **(A–B)**: bronchiolar columnar epithelium enveloped by a thin layer of smooth muscle cells (**arrows**); thin parenchymal septa line the alveoli. **(C–D)**: disorganized cylindrical epithelium with large intercellular spaces, and numerous goblet cells (**arrowhead**); thick septa and collapsed alveoli. **(E)**: disorganized epithelium; cylindrical cells have pale, secretory apical cytoplasm and lay on a hypertrophic smooth muscle layer (**arrows**); moderately thickened septa reduce the alveolar volume. **(F)**: quasi-columnar epithelium with dense cytoplasm, and moderately hypertrophic smooth muscle layer (**arrow**); thin septa and moderately collapsed alveoli. Inset: detail of the epithelium **(E)** and smooth muscle layer **(M)**. **(G)**: apparently normal airway with regular epithelium and smooth muscle layer **(arrow)**; thin septa and wide alveoli. Inset: detail of the epithelium **(E)** and smooth muscle layer **(M)**. Epithelium **(E)**, septa (**s**), alveoli **(A)**, connective fibers (*), airway lumen (l), vessel (v). Image acquisition with Leica System; Animal for each group *n* = 3; bars: 80 μm **(A, G)**, 50 μm **(B,C,E, and F)**, 25 μm **(D, inset)**.

Histological examination was performed on the airways in control mice (Figures 4A, B), and observed a columnar epithelium lining the airways, with densely packed cells having a basal nucleus (Figure 4B). Below the thin basement membrane, a bi/tri-layer of smooth muscle cells was present (Figure 4B). The parenchyma contained a net of thin septa, delimiting well-distended alveoli (Figures 4A, B s). Close to the large vessels, the connective thickened, and a bunch of fibers was often visible (Figures 4A, B). Following OVA sensitization (Figures 4C, D), significant changes occurred. The cells became cylindrical, separated by evident intercellular spaces, and the goblet cells were more evident, protruding into the lumen of the airway (Figure 4D). Marked changes also occurred at the level of the parenchyma: the septa were thick, and the alveoli collapsed (Figure 4C). Connective fibers were present, close to the large vessels, connecting the structure with the airway (Figure 4C). In mice treated with PS (Figure 4E), the epithelial cells became columnar, and the apical cytoplasm was irregular and secretory (Figure 4E). The smooth muscle layer was hyperplastic (Figure 4E), and, in the parenchyma, the septa were thick and delimiting alveoli moderately reduced in volume. After TBZ treatment (Figure 4F) the epithelium showed moderately elongated cells, with occasional intercellular spaces. The cell cytoplasm was uniformly dense, the nuclei were basal (Figure 4F, inset). The smooth muscle was not hypertrophic, the septa were moderately thickened, and the compressed alveoli were less numerous. The administration of PS-TBZ induced a significant recovery of the epithelium: the cells reduced in height, their cytoplasm returned dense and homogeneous, and the intercellular spaces were almost absent (Figure 4G, inset). The parenchyma also returned to normal, with thin septa and large, regularly arranged alveoli (Figure 4G).

### 3.4 PS-TBZ inhibits airway remodeling in OVA-sensitized mice

**FIGURE 5:**
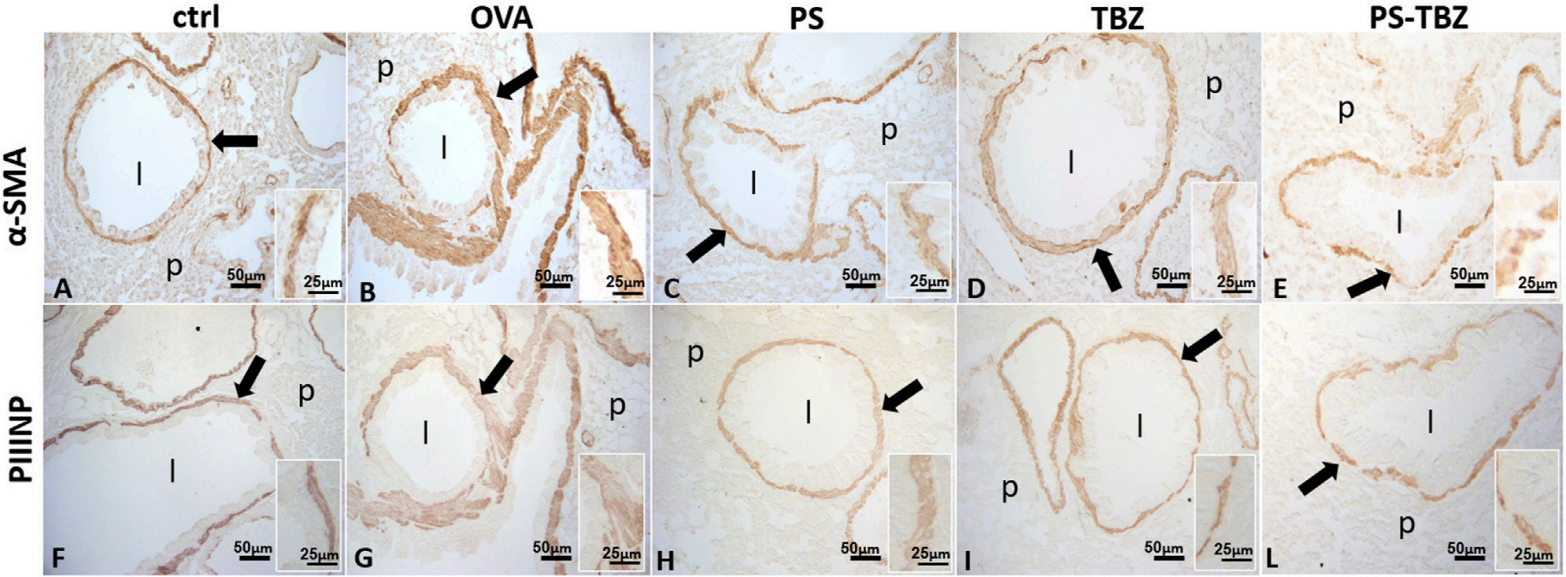
Effect of PS, TBZ, or PS-TBZ treatments on airway remodeling in sensitized mice. Expression of α-Smooth Muscle Actin (α-SMA; **A–E**) and type III Procollagen Peptide (PIIINP; **F–L**). Was assessed on lung sections as indicated by arrows. Inset: detail of the smooth muscle layer. Airway lumen (l), unstained parenchyma (p). Image acquisition with Leica System; group *n* = 3; bars: 50 μm (A–L), 25 μm (inset).

In the OVA-sensitized mice AHR was positively correlated with an increase in α-SMA expression (Figure 5B) which confirms the activation of myofibroblasts after OVA exposure, as further supported by increased procollagen III production (PIIINP, Figure 5G). PS treatment partially counteracted the remodeling process as indicated by the reduced α-SMA (Figure 5C) and PIIINP expression (Figure 5H) in the peri-bronchioles section. In the same way, TBZ treatment partially affected the remodeling process (Figures 5D, I) maintaining the typical elongated structure of myofibroblasts in the peri-bronchioles section. PS-TBZ treatment modified the typical elongated structure of myofibroblasts induced by OVA, and it reduced both α-SMA (Figure 5E) and PIIINP (Figure 5F) expression.

### 3.5 PS-TBZ mitigates the disorganization of peri-bronchiolesal collagen fibres

**FIGURE 6:**
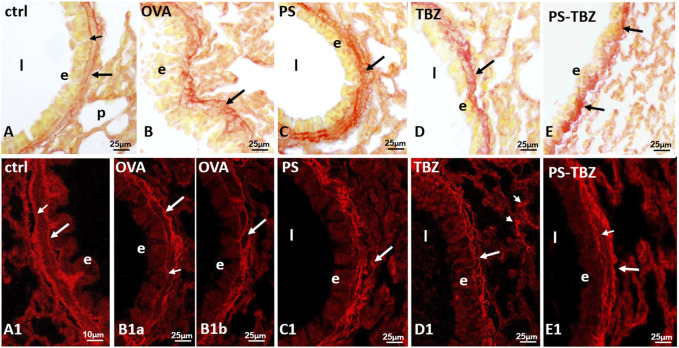
Effects of PS, TBZ or PS-TBZ treatments on collagen organization in lungs of sensitized mice. PSR staining was performed on lung sections. **(A, A1)**: bronchiole wall with stained basal lamina (**small arrow**) and a thin layer of collagen (**arrow**) enveloping the smooth muscle layer. **(B, B1a)**: thick and disorganized fibrils (**arrow**). **(B1b)**: area of almost regular appearance; basal lamina (**small arrow**) and collagen under the smooth muscle (**arrow**). **(C, C1)**: high directional dispersion of thick collagen fibrils (**arrow**). **(D)**: recompacted fibrils reform a sort of bilayer (**arrow**). Note in **C1** the presence of thick fibrils in septa (**arrowheads**). **(E):** compacted fibrils (**arrows**) from patches with regular bilayer **(E1)**. Airway lumen **(l)**, bronchiolar epithelium **(E)**. Image acquisition with Leica System **(A–E)** and confocal microscopy Zeiss LSM700 **(A1–E1)**; group *n* = 3; bars: 10 μm (A1), 25 μm (**A–E**, **B1a–E1**).

PSR staining was used to examine collagen distribution in various conditions. In the control group (Figures 6A, A1), collagen formed a continuous and uniform layer at the basal membrane level. A second, moderately irregular layer was observed at the base of the smooth muscle layer (indicated by a big arrow in Figure 6A). After exposure to OVA (Figure 6B), collagen fibers appeared thicker and more dispersed in a specific direction. The typical bilayer organization seen in controls (Figures 6A, A1) was less common following OVA exposure (Figures 6B, B1b), partly due to irregularities in the basal membrane (Figure 6B1A). Furthermore, PS exacerbated the situation, causing collagen fibers to become even thicker, curled, and highly disorganized, invading the smooth muscle region and extending into the underlying connective tissue (Figure 6C). The basal membrane was rarely recognizable (Figures 6C, C1). Following exposure to TBZ (Figures 6D, D1), collagen fibers compacted, forming dense amorphous patches at times (Figure 6D1). The administration of PS-TBZ resulted in further thickening of the fibrils in some areas (Figure 6E), while in other areas, an almost complete restoration of the bilayer organization was observed (Figure 6E1).

### 3.6 PS-TBZ inhibits mucin production

**FIGURE 7:**
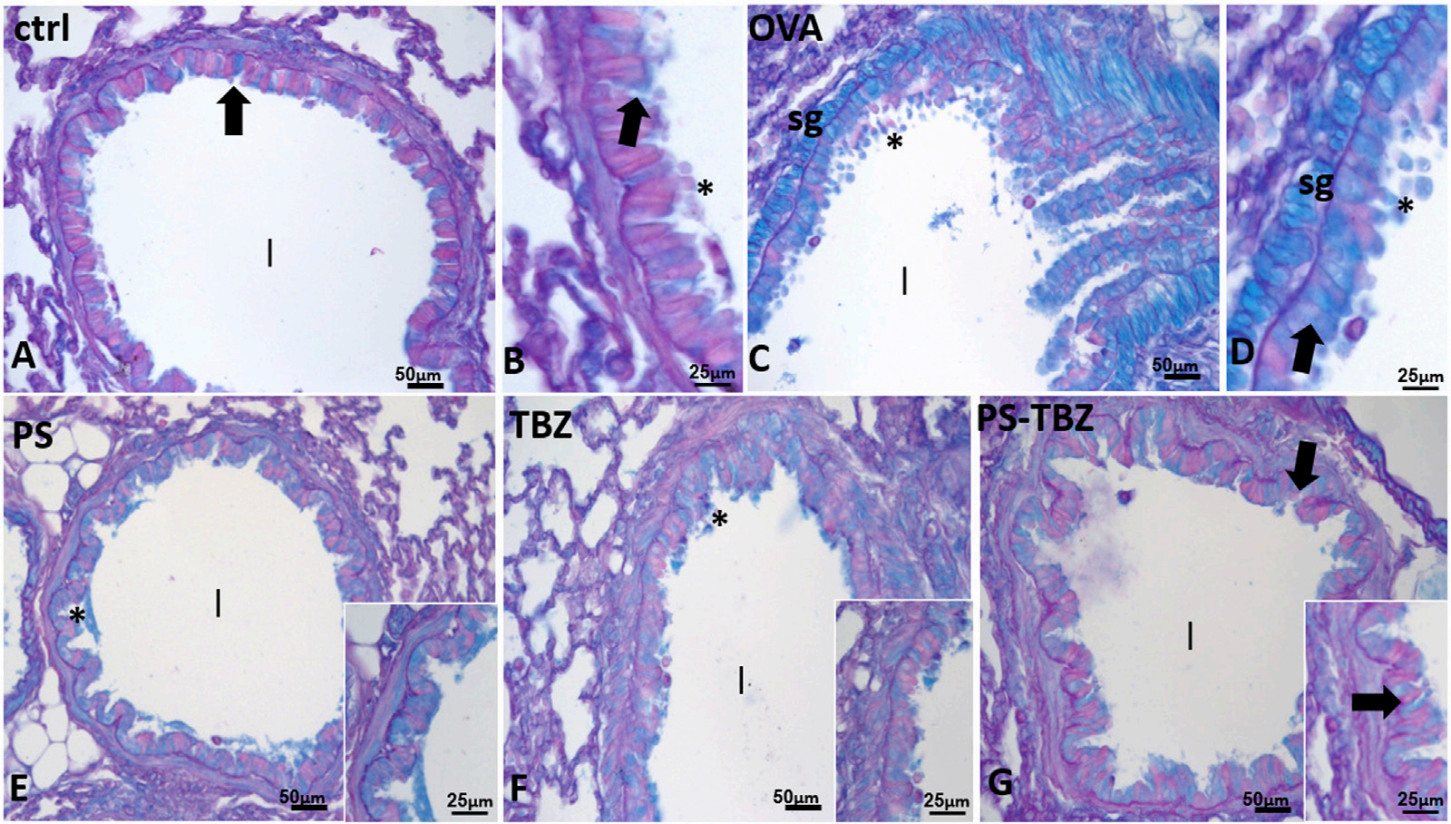
Effects of PS, TBZ, or PS-TBZ treatments on mucin production induced by sensitization. PAS^+^/AB was performed on lung sections. **(A, B):** Control airway with scattered goblet cells (**arrows**) and mucus (*) in the lumen (**l**). **(C, D):** increased number of goblet cells (**arrows**) and mucus (*) in the lumen. Note the positivity of submucosal glands (**sg**). **(E, G):** decreased number of positive epithelial goblet cells (**arrows**), no evident submucosal glands, and almost complete disappearance of mucus in the airway lumen (**l**). Image acquisition with Leica System; group *n* = 3; bars: 50 μm **(A,C,E,F, and G)**, 25 μm (**B, D**, inset).

PAS+/AB staining (Figure 7) was used to detect acid mucins. In the control group (Figures 7A, B), there was minimal mucus present. After exposure to OVA (Figure 7C), the presence of mucus significantly increased in peri-bronchial and epithelial goblet cells, leading to its release into the airway lumen (Figure 7D). PS (Figure 7E) and TBZ (Figure 7F) treatments reduced mucin levels at both locations, but the mucus in the airway lumen appeared denser and filamentous. When PS-TBZ was administered in combination (Figure 7G), the condition was almost restored to that of the control group, as observed following the single compound treatment, but the new chemical entity achieved the same effect with half dose Indeed, goblet cells were scattered in the epithelium, submucosal glands remained unstained, and very little mucus was observed in the bronchioles lumen (Figure 7G).

### 3.7 PS-TBZ modulates changes in carbohydrate composition induced by OVA sensitization

**FIGURE 8:**
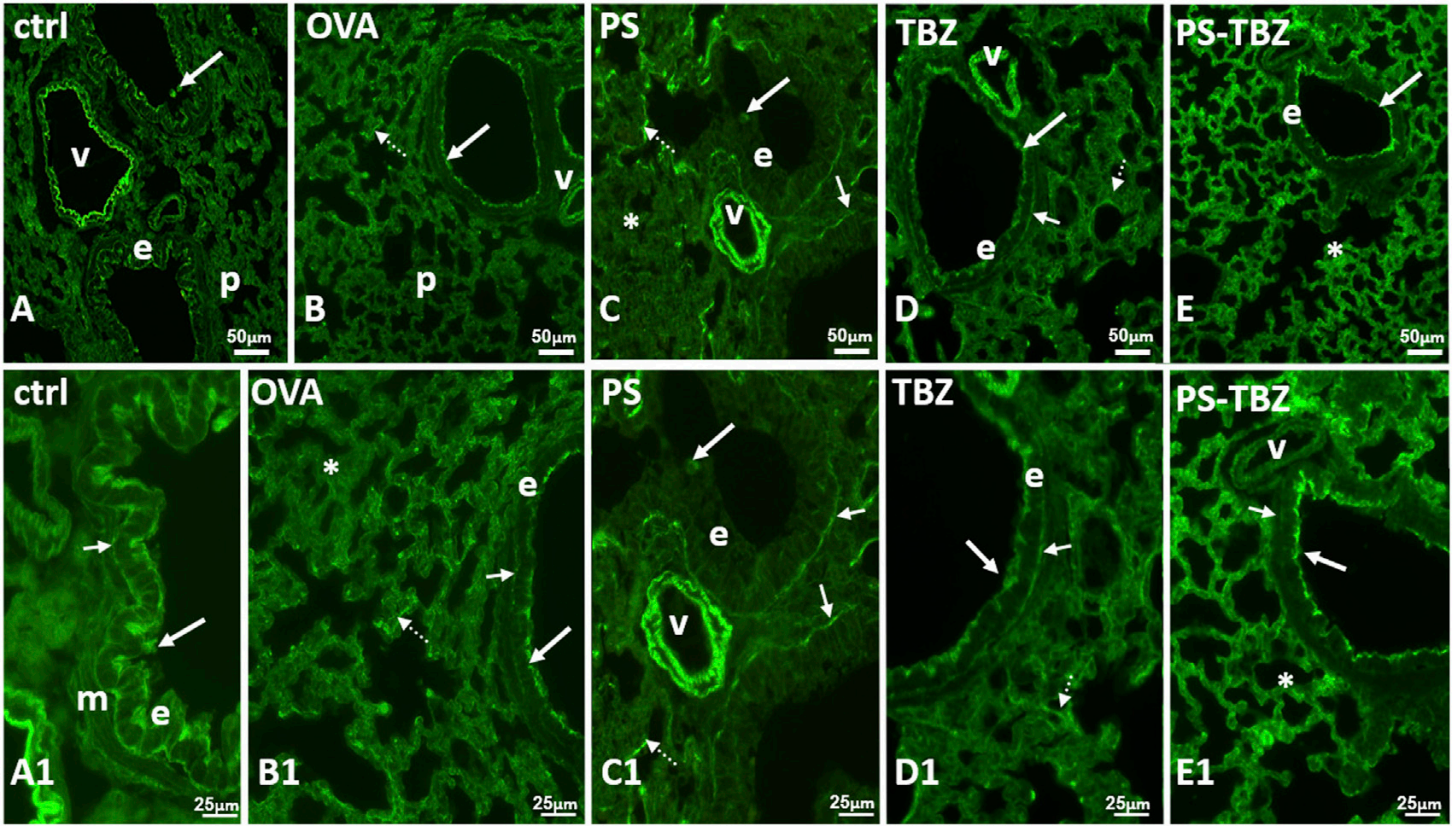
Effect of PS, TBZ, or PS-TBZ treatment on N-Ac-glucosamine distribution. **(A, A1)**: labeled elastic lamina of the vessels (**v**), apical cytoplasm (**arrows**) of the bronchiolar epithelium (**e**), and basal lamina (**small arrow**). Muscle (**m**) is unstained. **(B, B1)**: labeled apical cytoplasm (**arrow**) of the bronchiolar epithelial cells **(E)**. Notice the irregularity labeled basal membrane (**small arrow**) and the parenchymal septa containing labeled fibres (**dotted arrows**). Labelled basal membrane (**small arrows**), and fibres in parenchymal septa (**dotted arrows**). Unstained parenchyma (*). **(C, C1)**: unlabelled epithelial cells (**e**) with occasional stained mucus cells (**arrows**). Labeled basal membrane (**small arrows**), and fibres in parenchymal septa (**dotted arrows**). Unstained parenchyma (*). **(D, D1)**: labeled elastic lamina of the vessels (**v**), apical cytoplasm (**arrow**) of the bronchiolar epithelium (**e**), basal membrane (**small arrows**) and fibres in parenchymal septa (**dotted arrows**). **(E, E1)**: labeled apical cytoplasm (**arrows**) of the bronchiolar epithelium (**e**), and fibres in all parenchymal septa (*). Unlabeled basal membrane (**small arrows**) and vessels (**v**). Image acquisition with Leica System; group *n* = 3; bars: 50 μm **(A–E)**, 25 μm **(A1–E1)**.

Staining with WGA lectin (Figure 8) demonstrated that OVA-sensitization caused a decrease in N-Acetyl-glucosamine (glcNAc) in the apical cytoplasm of bronchioles epithelial cells and in the basal lamina. In contrast, sensitization significantly increased lectin positivity in goblet cells and, to a lesser extent, in alveolar septa (Figure 8B1). After PS treatment (Figures 8C, C1), labelled goblet cells were reduced while significant labelling was restored at the level of the basal lamina. No labeling however appeared on the epithelial apical cytoplasm (Figure 8C1), and alveolar septa remained labelled. TBZ (Figures 8D, D1) and PS-TBZ (Figures 8E, E1) restored labelling on the apical cytoplasm of the epithelial cells and on the basal membrane. TBZ alone reduced labelling in the alveolar septa while the combination determined a significant increase in such labelling (Figure 8E). Unexpectedly, vessel walls that were always labelled (Figures 8A1, 8C1) after PS-TBZ appeared completely unstained (Figure 8E1).

## 4 Discussion

Corticosteroid use in asthma in controlling inflammation is limited by the side effects and by insensitivity in some people (Cazzola and Hanania, 2006; Bousquet et al., 2007; Kuna et al., 2007; Heffler et al., 2018). It is becoming increasingly clear that several airway diseases are associated with a state of H_2_S deficiency (Chen et al., 2009a; Chen et al., 2009b; Bartman et al., 2020; Hughes et al., 2009). We have previously demonstrated the ability of H_2_S donors such as NaHS to modulate bronchial tone and reverse AHR (Roviezzo et al., 2015). In this study we investigated the therapeutic efficacy of a new corticosteroid derivative, namely, PS-TBZ, that can slowly release H_2_S. We demonstrate that this hybrid could provide a suitable approach to manage asthma feaures with a particular impact on airway remodeling.

Acute and chronic respiratory diseases constitute a major health burden in both children and adults (Hammad and Lambrecht, 2021; Hizawa, 2023). Asthma affects ∼300 million people globally and contributes to 1 in 250 annual deaths (Maciag and Phipatanakul, 2020). The pathogenesis of asthma involves three main components: airway inflammation, airway remodeling, which refers to structural changes in the airways that eventually lead to airway fibrosis and obstruction, and AHR, representing the clinical symptom of asthma (Hough et al., 2020; Hammad and Lambrecht, 2021). Current therapies primarily target inflammation and bronchoconstriction (Van Den Berge et al., 2009; Gabehart et al., 2013), but airway remodeling can develop also independently of inflammation, limiting the efficacy of pharmacological treatments (Gabehart et al., 2013; Hough et al., 2020). Thus, a reduced or lack of efficacy on remodeling, that leads to a decline in lung function, represents an important limitation in pharmacological therapy (Bergeron et al., 2010).

To overcome the limitations of current therapies, we explored the possibility to chemically combine H_2_S donors with prednisone. For this purpose, we have chosen a slow H_2_S donor such as TBZ. The PS-TBZ can release discrete amounts of hydrogen sulfide (Corvino et al., 2021a) and displays a significantly improved pharmacological profile, as demonstrated by the *in vitro* experiments. Both PS and TBZ exhibited a concentration-dependent anti-inflammatory activity as indicated by the decreased production of LPS-induced NO, IL-1β, PGE_2_, and TNF-α. In the same experimental conditions, PS-TBZ clearly showed an enhanced antinflammatory action. TBZ exhibited also a weak bronchial relaxant activity and counteracted bronchial reactivity to carbachol. Therefore, TBZ extends the therapeutic efficacy of prednisone in terms of anti-inflammatory action and adds new effect as the ability to directly relax airway smooth muscle. This means that the hybrid compound can achieve a therapeutic effect with smaller doses. This could also translate into a reduction of the risk of side effects associated with the prolonged use of corticosteroids. In addition, PS-TBZ exhibits great chemical stability at two pH conditions (1.2 and 7.4) and it is enzymatically hydrolyzed in human serum, resulting in a successful hybrid (Corvino et al., 2021a).

Based on these findings we conducted *in vivo* experiments using a mouse experimental model of asthma. PS, TBZ, or PS-TBZ have been administered during the sensitization phase. Bronchi harvested from sensitized mice showed increased bronchial reactivity to carbachol that is partially restored by PS treatment. Conversely, TBZ or PS-TBZ completely prevented allergen-induced AHR. Thus, PS-TBZ exhibited the combined pharmacological activities of PS and TBZ, effectively counteracting AHR with lower doses of both drugs. The allergic inflammatory reaction induced by sensitization also causes impairment in β2-adrenoceptor-mediated relaxation induced by heterologous desensitization. This represents an important limitation of bronchodilator therapy since inhaled selective short-acting β_2_ agonists such as salbutamol, are the rescue treatment of choice for the relief of symptoms of acute asthma. Pretreatment with PS-TBZ preserved salbutamol-induced relaxation as well as TBZ, while PS alone had no effect. This means that PS-TBZ shows additional effects in controlling airway dysfunction taking advantage of TBZ action, with a better therapeutic profile than PS alone.

Airway dysfunction correlated with IgE-mediated inflammation, as indicated by increased plasma IgE levels. PS administration as expected significantly reduced IgE increase, while TBZ had no effect. Although this effect was lost by PS-TBZ, this does not prejudice its efficacy in preserving airway dysfunction. It is plausible that the lack of effect on IgE is most likely due to the lower amount of PS when coupled with TBZ. The functional synergic effect is sustained by the different molecular mechanisms of PS and TBZ. Thus PS-TBZ acquires a new pharmacological profile with an enhancement of the pharmacological properties of the parent compounds. Histological evaluation of pulmonary sections further demonstrates the enhanced beneficial effects of PS-TBZ treatment. Indeed, allergen sensitization profoundly changes the epithelium structure, and this is an important step in triggering subepithelial mechanisms driving the airway remodeling process. The cells become cylindrical, separated by evident intercellular spaces, and the goblet cells are more evident, protruding into the lumen of the airway. The subepithelial smooth muscle layer appears moderately hypertrophic, appearing bi/tri-layered. Marked changes also occur at the level of the parenchyma as evident by alveoli collapse. PS or TBZ treatment partially restored the bronchial epithelium, but PS-TBZ treatment induced a more complete recovery of the epithelium and parenchymal structure.

An active remodeling process is evidenced also by α-SMA expression upregulation following sensitization. The activated state of fibroblasts was confirmed by the increased procollagen III production. PS and TBZ treatments partially affected the remodeling process.In contrast, PS-TBZ significantly reduced the remodeling process compared to treatments alone. The smooth muscle in sensitized mice is slightly thicker than the control, and treatment with PS worsens the situation. In histochemistry pictures α-SMA indicates the conversion of fibroblast into myofibroblast, and, even if the smooth muscle in PS is thicker, fibroblasts are not all activated in myofibroblasts and consequently the production of collagen is lower than treatment with allergen. Mice receiving PS or TBZ treatment also showed a significant reduction in acid mucins induced by allergen exposure. PS-TBZ exhibited a similar protective effect to the parent compounds but at a 50% lower dose on a molar basis. This effect exhibited by PS-TBZ is also evident on the whole bronchi as evidenced by lectin staining. The loss of lectin represents a serious event, for any type of cell since it plays fundamental roles in the extracellular matrix and in cell signaling (Konopka, 2012). In the lungs, it has been related to airway inflammation (Krick et al., 2018). Following sensitization collagen fibers frequently appeared thickened and/or showed a high directional dispersion. The typical bilayer organization seen in controls was very hardly visible due to the frequent irregularities in the basal membrane. PS worsened the situation: collagen fibers became thicker, curled, and markedly disorganized, to invade the smooth muscle region, often extending in the underneath connectives and the basal membrane was rarely recognizable.TBZ induces a good recovery in the epithelium, in line with the morphological aspect and, importantly, also of positivity in the basal lamina. This is essential for the proper functioning of the epithelium. The administration of the PS-TBZ hybrid caused an almost complete restoration of the bilayer.

In conclusion, our findings further demonstrate the essential role of H_2_S in airway function and its contribution to improving asthma features. Moreover, we have demonstrated that PS-TBZ displays a favorable therapeutic profile *in vivo* with some additional effects. Future studies will be necessary to further characterize the molecular mechanisms underlying the beneficial effect of PS-TBZ and in particular the role of TBZ. However, this combination provides an opportunity to optimize the beneficial impact of corticosteroids by reducing dosages and associated side effects.

## Data Availability

The original contributions presented in the study are included in the article/Supplementary Material, further inquiries can be directed to the corresponding authors.
